# COVID-19 and communication: A sentiment analysis of US state governors’ official press releases

**DOI:** 10.1371/journal.pone.0272558

**Published:** 2022-08-30

**Authors:** Mauricio Tano, Juha Baek, Adriana Ordonez, Rita Bosetti, Terri Menser, George Naufal, Bita Kash

**Affiliations:** 1 Nuclear Engineering Department, Texas A&M University, College Station, TX, United States of America; 2 Department of Health Care Policy Research, Korea Institute for Health and Social Affairs, Sejong-si, Korea; 3 Department of Population Health Sciences, Weill Cornell Medical College, New York, NY, United States of America; 4 Public Policy Research Institute, Texas A&M University, College Station, TX, United States of America; 5 Department of Health Policy and Management, School of Public Health, Texas A&M University, College Station, TX, United States of America; University of Edinburgh, UNITED KINGDOM

## Abstract

**Objectives:**

This study examines the contents of official communication from United States governors’ offices related to the COVID-19 pandemic to assess patterns in communication and to determine if they correlate with trends for COVID cases and deaths.

**Methods:**

We collected text data for all COVID-19 related press releases between March 1 and December 31, 2020 from the US governors’ office websites in all 50 states. An automated parsing and sentiment analyzer assessed descriptive statistics and trends in tone, including positivity and negativity.

**Results:**

We included a total of 7,720 press releases in this study. We found that both positive and negative sentiments were homogenous across states at the beginning of the pandemic but became heterogeneous as the pandemic evolved. The same trend applied to the frequency and tone of press releases. Sentiments across states were overall positive with a small level of negativity. We observed a reactive official communication to the evolution of the number of COVID-19 cases rather than responsive or preventive.

**Conclusions:**

The findings of both positivity and negativity in press communications suggest that the effect of discounted importance was present in official communications. Our findings support a state-dependent optimal communication frequency and tone, agreeing with the curvilinear communication model of organizational theory and implying that feedback cycles between government officials and public response should be shortened to rapidly maximize communication efficacy during the pandemic. Future research should identify and evaluate the drivers of the large differences in communication tone across states and validate the reactive characteristics of COVID-19 official communications.

## Introduction

The COVID-19 pandemic has affected all populations—causing physical and mental health issues—and the economy in the United States (US) and around the world [1–3]. The pandemic also generated a tremendous flow of information as science and medical research better understood SARS-CoV-2. The White House Coronavirus Task Force conducted daily press briefings to provide guidance throughout the pandemic [4]. Concurrently, state governors provided updates and guidelines to their constituencies by using regular briefings [5,6]. General press and social-media influencers based their communications on official press releases but also contributed to fostering an environment of misinformation that could have increased social tensions and discriminatory behaviors [7–9]. People have especially used social media to seek health information and social support during the pandemic and to share updated information related to COVID-19 [10,11]. Thus, official communications had to convey accurate information and transmit hopeful, consistent, and positive messages for mitigating population uncertainty, discrimination, and social tensions [4].

Communications from official leadership during an emergency situation play a significant role in crisis management and successful implementation of rapid responses [12]. Previous studies investigated the US state governors’ press releases during the COVID-19 pandemic to evaluate the effectiveness of stay-at-home recommendations and communication styles among different governors [6,13]. However, none of these studies has evaluated patterns of governors’ official statements over time during the pandemic.

Effective communication between leadership and the public, in terms of transparency and confidence, is essential to manage public health crises since communication is the basis of trust, thereby influencing individuals’ and communities’ compliance with the recommended behaviors [7–9,12,14,15]. Moreover, the tone or sentiment of a message is a key factor which could influence the perception of transparency and confidence [16,17]. However, there is a lack of case-driven studies determining how tone should be regulated in official communications during the pandemic. Therefore, we performed a retrospective study on the tone of the US state-level official communications during the COVID-19 pandemic by using a sentiment analysis, adding knowledge that could guide the tone of official communications in future critical situations.

Sentiment analysis via machine learning provides a structured, systematic, and unbiased procedure for information modeling [18]. Changes in sentiment over time can be associated with information inconsistency [19]; by correlating these changes to events this technique can measure awareness and emotional reactivity [20]. By using sentiment analysis of official press releases from the highest leadership in each state, this study aims to examine the contents of official communication from US governors’ offices related to the COVID-19 pandemic to assess patterns in communication and to determine if the patterns correlate with the trends for COVID cases and deaths. Specifically, this study had four main objectives:

1. To examine how the tone (positivity and negativity) in official press releases have evolved during the COVID-19 pandemic.
2. To evaluate the magnitude of fluctuations in positivity and negativity in official press releases across states when compared to the national averages.
3. To investigate how the evolution of tone was related to the change in the numbers of COVID-19 cases and deaths per state.
4. To examine any correlations between frequency/sentiment of press releases and governor/state characteristics.

This study calls attention to communication and messaging, which are important components of dealing with a pandemic, which is very different than natural disasters. Disasters are often short lived and less widespread, affecting specific regions for a certain amount of time. COVID-19 has been affecting every region for an extended and indefinite period, so understanding how official communications differ across states and over time allows us to identify areas of improvement in communication in time for future potential pandemics. Questions about the homogeneity of official messaging and their tone can also ground additional research into whether such events are linked to responses from people and to the evolution of the pandemic. As such, our discussion below includes a special emphasis on policy recommendations.

## Materials and methods

### Study design and data collection

We searched for COVID-19 related press releases through governors’ official websites. This study included only state governors’ press releases and excluded communications from the State Health Department and executive orders, which frequently presented a more legal and formal tone. We collected a total of 7,720 press releases with >36 million words. The data included COVID-19 related press releases from all 50 state governors’ offices between March 1, 2020 and December 31, 2020. The period coincides with the first ten months of the spread of the COVID-19 virus in the US and ends two weeks after the first vaccine shot was given.

**Fig 1:**
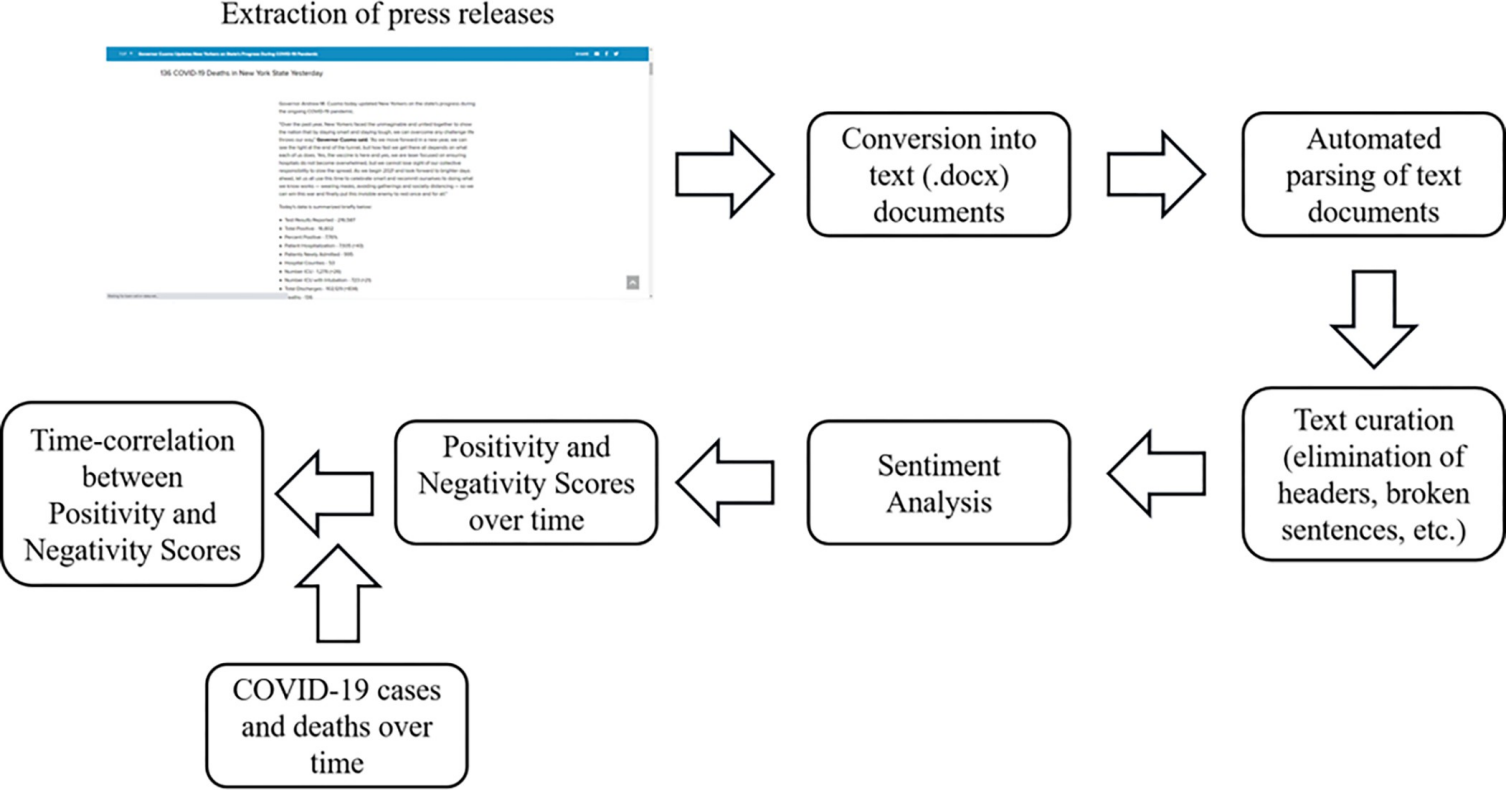
Processing pipeline to analyze the sentiment in governors’ official press releases over time and to correlate them with the numbers of state COVID–19 cases and deaths.

Fig 1 illustrates the processing pipeline of this study. Each press release constituted one observation. When there was more than one press release available in one day, we created a differentiating label. We saved each press release in a separate Microsoft Word document and automatically parsed using the doc2txt v0.6 library in Python programming language. We automatically removed extra headers and footers copied into the Word document file by keyword identification (e.g., weblink) and spatial information (e.g., spaces at the beginning of the end of the text). Then we fed each parsed file into a sentiment analyzer (see below) and computed the correlation between positivity and negativity scores with the numbers of COVID-19 cases and deaths.

### Description of press releases

We computed the total number of press releases, total number of words in all press releases, and average density of words per article for each state during the study period (for detailed information refer to S1 Appendix). We normalized the number of articles per state by the total number of published articles and computed the monthly frequency of articles published per state.

### Sentiment analysis

To face the lack of adapted training data when building a sentiment analyzer, we designed a sentiment analyzer architecture inspired by one recently proposed by Behera et al. [21] A detailed explanation of the architecture of our sentiment analyzer is provided (see S2 Appendix). The proposed architecture has two key advantages. First, by using the correlation matrix of vectorized text segments, the output of the network is less dependent on the specific context in which positivity and negativity are classified. The sentiment analyzer was trained in standard sentiment classification dictionaries, and its accurate performance on the press releases was assessed by inspecting the scores assigned to 1,000 randomly selected outputs. Second, an attention mechanism allows the neural network to integrate *common sense* knowledge and output biases and aliasing, which was helpful when modifying the context for applying the neural network. As shown in Fig 1, during the analysis, we fed each tokenized press release into the sentiment analyzer. The sentiment analyzer sequentially parses the text word by word until a sentiment can be assigned with statistical confidence to the token.

### Longitudinal and cross-sectional analyses of press releases’ sentiment

A sentiment analyzer evaluated all press releases. The output of this process was a probability density function indicating the positivity and negativity scores for all the tokens identified for each text. These scores were grouped by state and month, and the ensemble probability density function was computed as the product of the individual probability density functions of all articles in that state and month. We used the pseudo-marginal Metropolis-Hastings Markov chain Monte Carlo algorithm [22] to compute the ensemble distributions of probability. We analyzed the evolution in time of the most probable positivity and negativity per state. Then, most-probable monthly positivity and negativity were ensemble-averaged for all states to compute the monthly national average positivity and negativity scores. Identifying these rates allowed us to quantify the tone of the official message from governors during a time of crisis and uncertainty. Finally, the most probable positivity and negativity per state were ensemble-averaged in time to obtain the state-level average (with 25–75% confidence intervals and min-max values) positivity and negativity scores during the study period.

### Comparing the evolution of positivity and negativity with the change in COVID-19 cases and deaths

The research team obtained daily, state-level evolution in the numbers of COVID-19 cases and deaths signals from the COVID-19 Data Repository by the Center for Systems Science and Engineering (CSSE) at Johns Hopkins University [23]. We smoothed noise due to missing daily reports by using a third-order Savitzky–Golay filter with a three-day window size and computed their first and second time-derivatives using second-order central differences. Then, we conducted the Pearson’s correlation coefficients between the numbers of cases and deaths and their first and second time-derivatives against the positivity and negativity scores for each press release. A large absolute value of the Pearson’s correlation between sentiment and the signal indicates communication responsiveness, whereas a large correlation with the signal’s first and second derivative indicates reactivity and predictivity, respectively [21]. Finally, we computed the national average (with 5% and 95% confidence intervals) Pearson’s correlation coefficients by using unweighted average correlations per state and averaging the correlations for all the states. The signals for the numbers of cases and deaths were lagged in time by 5 days—determined via a sensitivity analysis seeking the highest correlation—when computing Pearson’s correlations.

### Differences in the frequency/sentiment of press releases by governor and state characteristics

As an obvious extension to the current analysis, we examined differences in the frequency and sentiment of press releases by governor and state characteristics. For this analysis, we chose as variables to characterize the sentiments per state the mean positivity and negativity of press releases, mean negativity of press releases, standard deviation in the positivity and negativity of press releases, number of press releases, and total number of words across all press releases. As variables characterizing the governor and state characteristics, we included the following governors’ attributes: political party affiliation (Republican or Democratic Party), gender (male or female), age (years), incumbency (years as a governor) and the state characteristics as follows: state population, geographical size, state budget in 2019 and 2020, and duration between lockdown and reopening (days).

## Results

### Description of press releases

**Fig 2:**
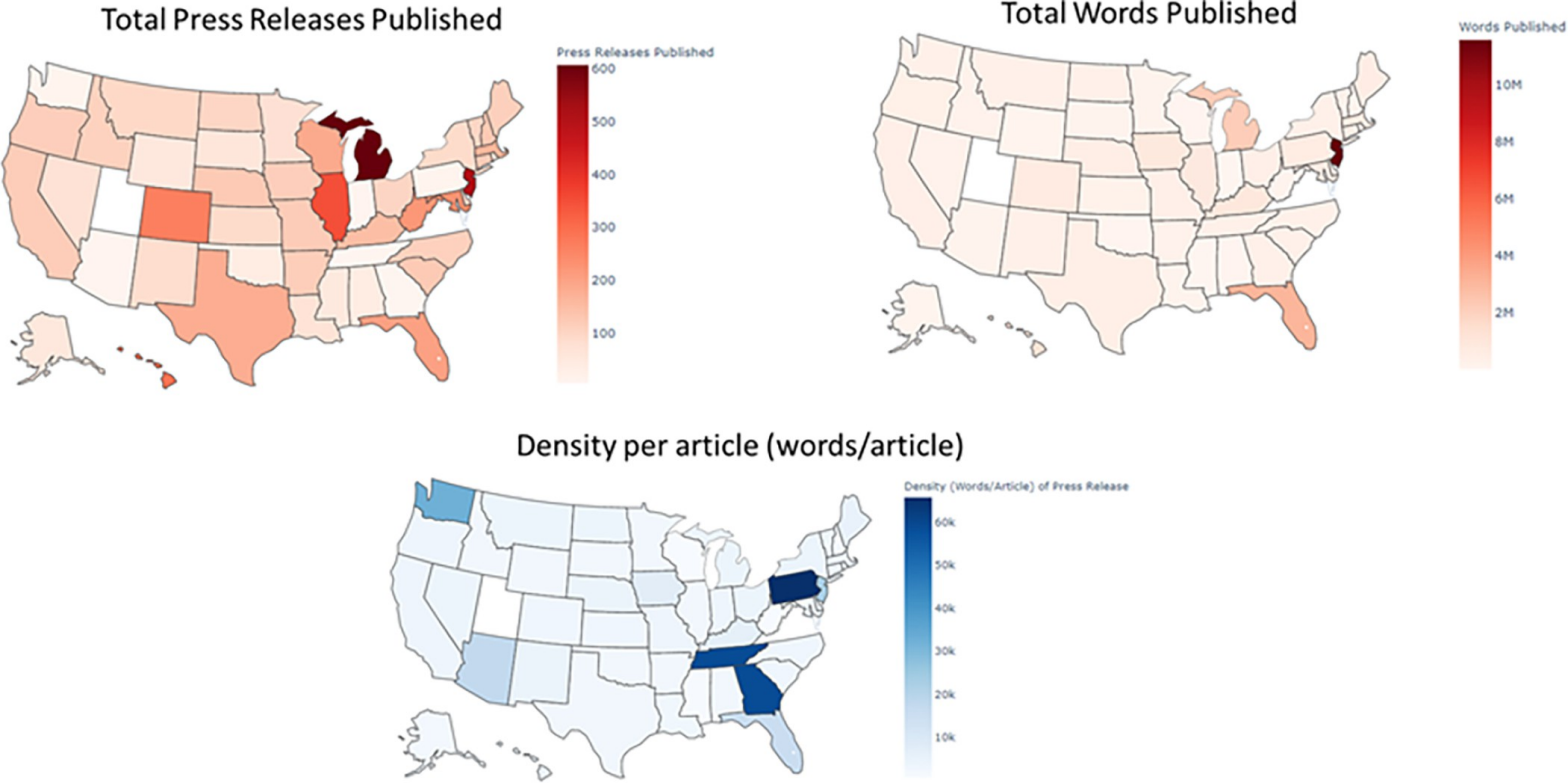
Comparison of total press releases, number of words, and density of words per article for all US states.

Fig 2 shows the total number of press releases, total words published, and word density per article for all states. We observed that more populous states released more information than smaller states. The amount of information released in terms of articles and words published was larger in the Northeast quadrant of the US, while the density of each press release was larger in the Southeast quadrant. This indicates two dissimilar communication strategies during the pandemic. Some states chose to give concise, frequent press releases, whereas others opted for less frequent but longer communications (For a list of scatter plots see S4 Appendix).

**Fig 3:**
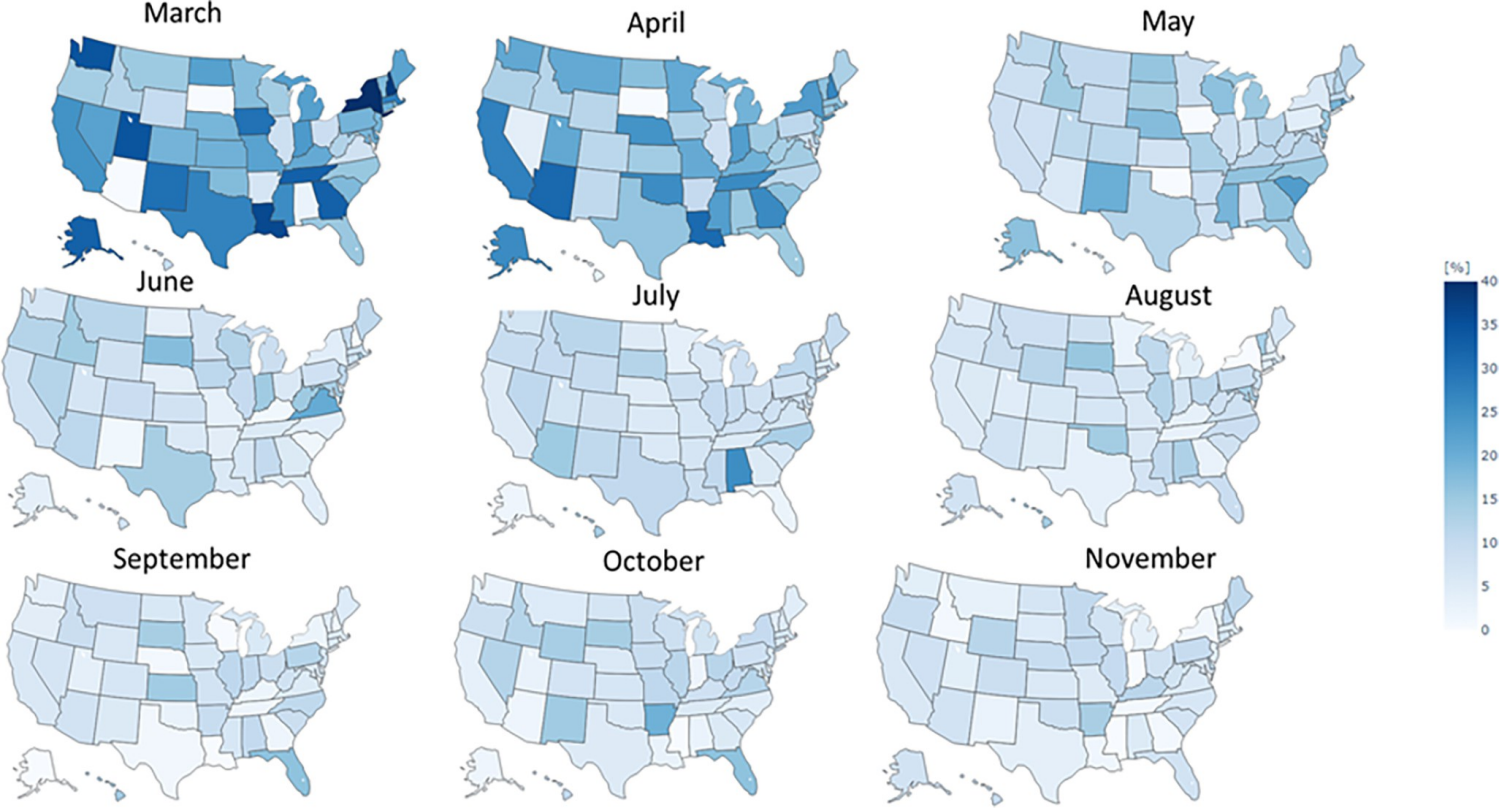
Normalized frequency of press–releases per state.

Fig 3 presents the state-normalized evolution of press releases over time. We found that most states provided more frequent press releases at the beginning of the pandemic (in March and April 2020) with reduced frequency thereafter, although localized peaks can be observed in some states after April 2020. Analyses on individual states showed that these peaks coincide with sudden rises in COVID-19 cases.

### Sentiment evolution during the pandemic

**Fig 4:**
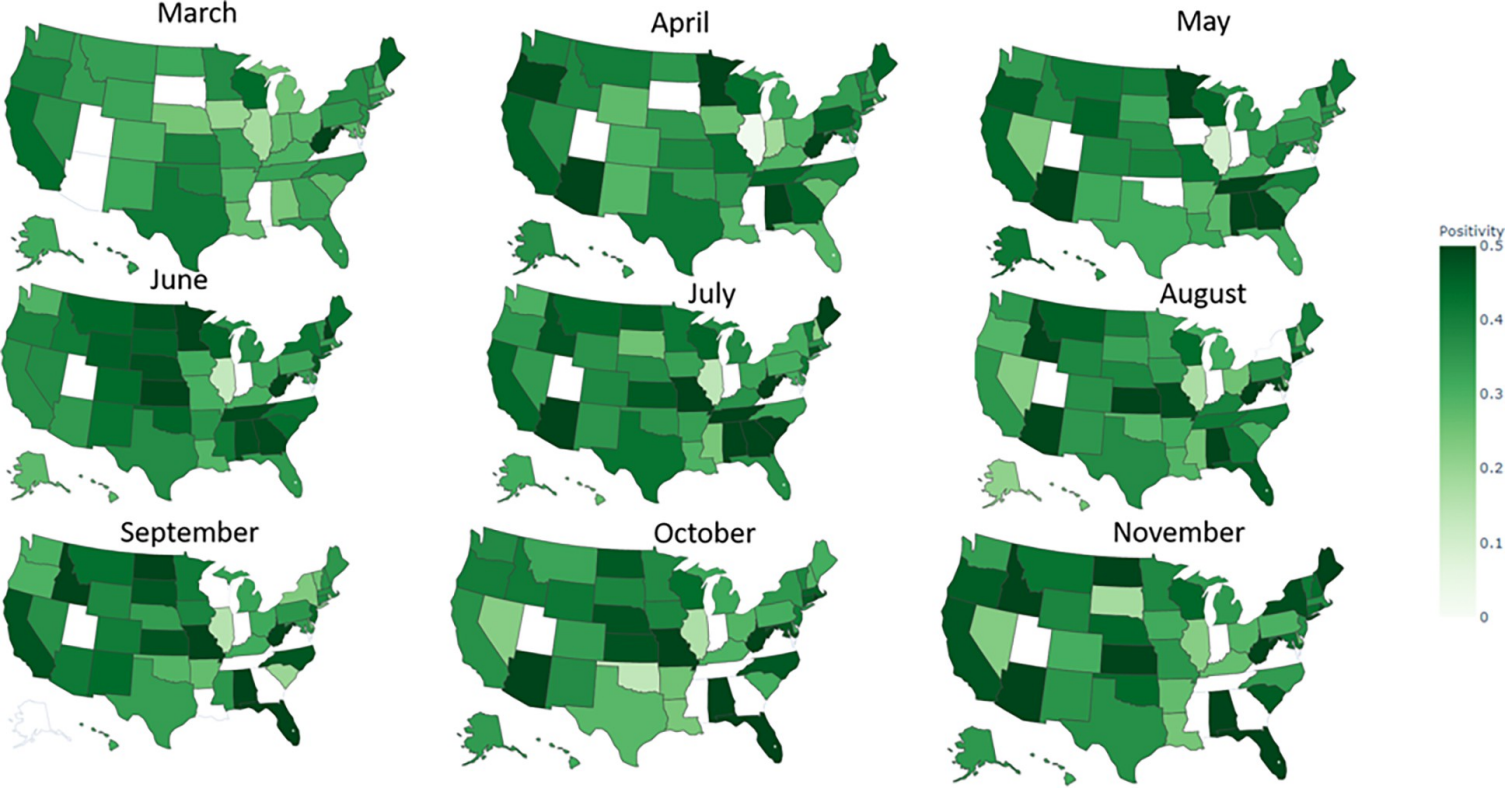
State–level evolution of tone (sentiment) during the study period: Evolution of positivity.

**Fig 5:**
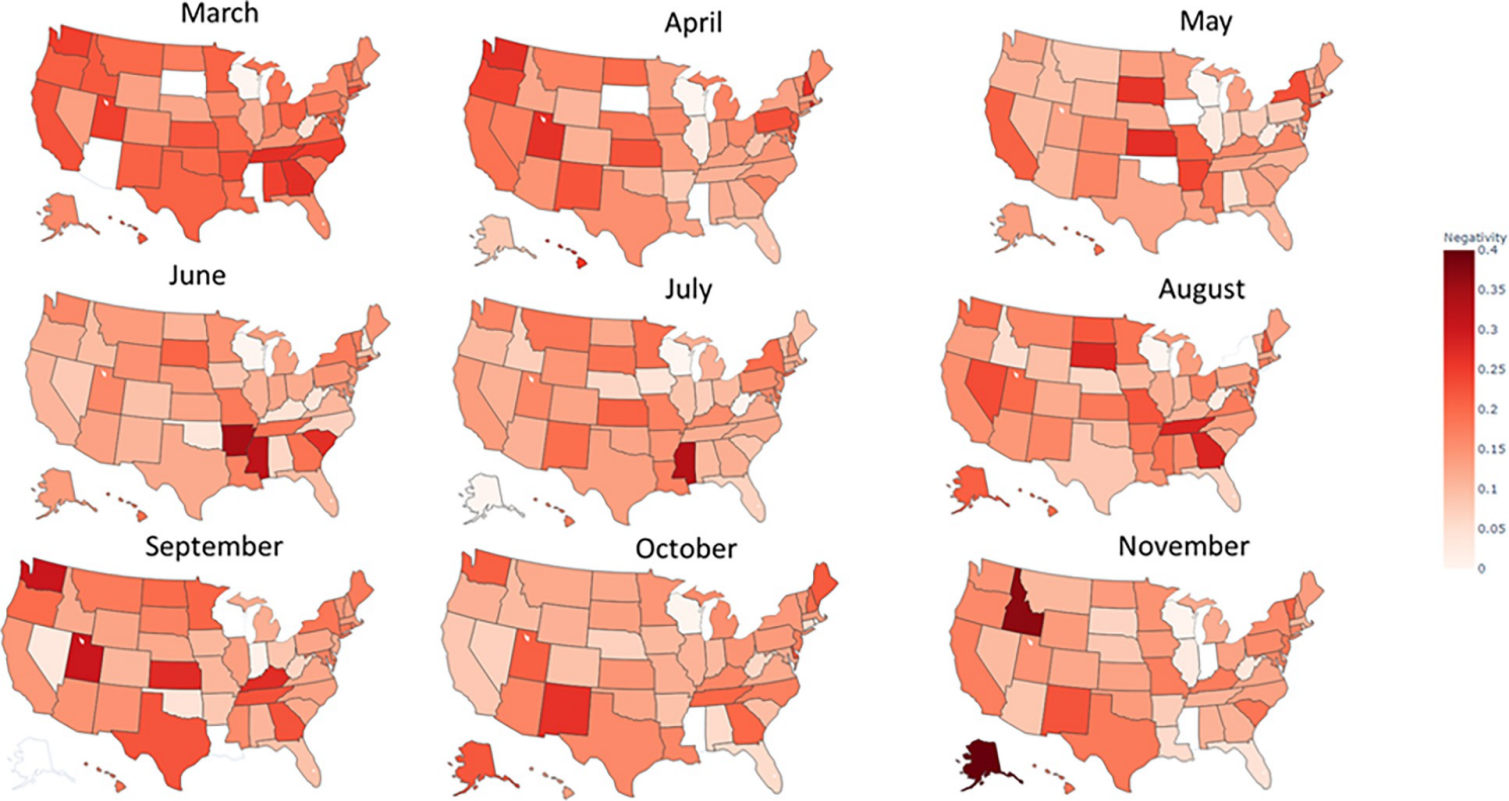
State–level evolution of tone (sentiment) during the study period: Evolution of negativity.

Figs 4 and 5 present the monthly, state-level evolution in positivity and negativity of press releases, respectively. We observed a general state polarization as the pandemic evolved, equating to both increased differences in positivity and negativity across states’ scores over time. Interestingly, positivity and negativity seemed to reach a state-dependent equilibrium value as the pandemic progressed. Contrary to frequency distribution, we did not find an outstanding geographical distribution for polarity.

**Fig 6:**
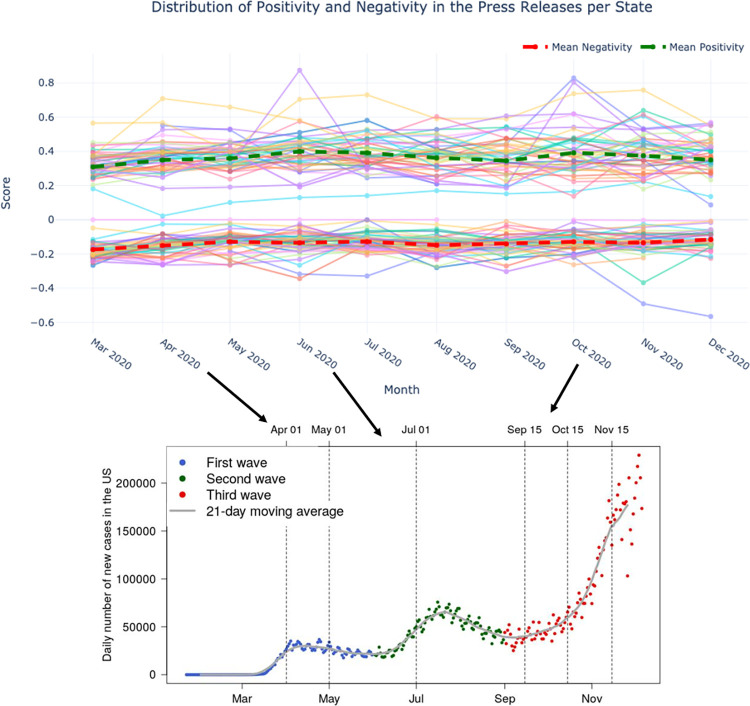
Positivity and negativity per state and national averages, and comparison with the COVID–19 waves.

Fig 6 evaluates state-level and state-averaged comparisons of the evolution of the positivity and negativity scores. As noted, we observed an average polarization during the pandemic for the state-average monthly evolution in positive and negative sentiment. Thus, positive and negative scores across states dispersed during the pandemic. Additionally, positivity increased and negativity decreased at the onset of each COVID-19 wave. Nonetheless, these national-level fluctuations were small when compared to the variance in positivity and negativity across states. As for state-level analyses, positivity and negativity also reached an equilibrium value as the pandemic progressed.

### Comparison of sentiment evolution across states

**Fig 7:**
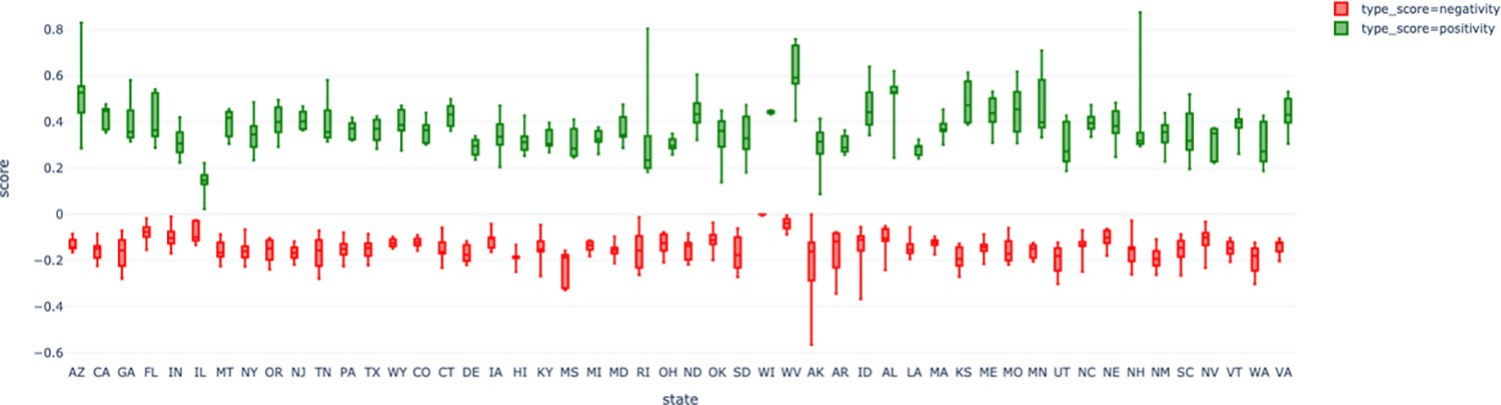
Average (standard deviation and 95% confidence intervals) for positivity and negativity per state during 2020.

Fig 7 compares the time-averaged (with 95%-CI and min-max values) positivity and negativity for all states during the study period. As Figs 4 and 5 show, communication sentiments varied substantially across states. Inter-state variations were still significant when compared to intra-state, adding to the evidence that communication strategies during the pandemic were independently tuned at the state level.

### Correlation between sentiment analysis and evolution in the numbers of COVID cases and deaths

Table 1 presents the results of the Pearson’s correlation coefficients of positivity and negativity and their first and second time-derivatives with the evolution of the numbers of COVID-19 cases and deaths. Regarding responsiveness, positivity increased and negativity decreased as both the numbers of COVID-19 cases and deaths increased. Nonetheless, the small value of these correlation coefficients indicates a large amount of dispersion in responsiveness. When analyzing reactivity, we observed higher correlations for the first time-derivatives of positivity and negativity with the numbers of COVID-19 cases and deaths. Finally, we found the lower correlations for the second derivative of sentiments with the numbers of COVID-19 cases and deaths, indicating that changes in positivity and negativity were, in general, non-preventive.

**Table 1:** Pearson’s correlation coefficient (5% and 95% confidence intervals in square brackets) between the smoothed rate of change in the numbers of COVID–19 cases and deaths and positivity and negativity of press releases and their first and second time derivative.

Metric	Number of COVID-19 cases	Number of COVID-19 deaths
**Positivity**	0.182 [0.026,0.338]	0.201 [0.036,0.366]
**Negativity**	-0.133 [-0.258,-0.008]	-0.120 [-0.236,-0.004]
**dPositivity/dt**	0.461 [0.294,0.628]	0.440 [0.264,0.616]
**dNegativity/dt**	-0.300 [-0.494,-0.106]	-0.287 [-0.479,-0.095]
**d2Positivity/dt2**	0.244 [0.062,0.426]	0.355 [0.160,0.550]
**d2Negativity/dt2**	-0.232 [-0.410,-0.054]	-0.252 [-0.436,-0.068]

### Correlation between frequency/sentiment of press releases and governor/state characteristics

Table 2 shows the results of the correlations between frequency/sentiment of press releases and governor/state characteristics. Republican governors were more likely to be positive in their press releases relative to their Democrat counterparts. Variations in the sentiment (either positive or negative) across press releases were larger for Democrats relative to Republicans. Democrat governors had more frequent press releases and the larger number of releases than Republican governors on average. We did not observe meaningful correlations regarding gender although female governors seem to be slightly more positive than their male counterparts. Age was only correlated with the mean positivity, with older governors being more positive on average. Governors start and end dates were not correlated with any of the variables.

**Table 2:** Result of correlation analysis between frequency/sentiment of press releases and governor/state characteristics.

	Mean positivity of press releases	Mean negativity of press releases	SD in positivity of press releases	SD in negativity of press releases	Number of press releases	Total number of words
**Governor characteristics**						
** Party**	0.16	0.04	0.28	0.25	-0.30*	-0.18
** Gender**	-0.13	0.08	-0.02	0.03	-0.03	0.03
** Age**	0.27	0.08	-0.07	-0.03	-0.15	-0.07
** Start date**	0.00	0.03	0.10	0.14	-0.02	0.03
**Experience** **(years)**	-0.00	-0.03	-0.10	-0.14	0.02	-0.03
**State characteristics**						
**Population**	-0.02	0.10	-0.21	-0.18	0.19	0.15
** Size**	-0.10	-0.25	0.03	0.61**	-0.14	-0.13
** Budget (‘19)**	-0.01	0.06	-0.25	-0.18	0.16	0.12
** Budget (‘20)**	-0.01	0.05	-0.24	-0.17	0.15	0.11
**Duration of** **lockdown** **(days)**	-0.09	-0.16	-0.18	-0.13	0.04	0.06

Note: SD–Standard Deviation

* p<0.05

** p<0.001.

In terms of state characteristics, the results showed that the budget was not correlated with any of the variables. Larger state populations showed less variation in sentiment (both positive and negative) and larger number of press releases and number of words. The opposite finding was true for the geographical size of the state, as the larger states showed a larger variability in the sentiment. Finally, the larger the duration between lockdown and reopening, the less polarization (so less positivity and negativity in the messaging tone of the press release) and smaller variations in the message (so more consistency). As expected, the longer the lockdown period, the larger the number of press releases.

We also conducted multiple linear regression to check robustness on the correlation matrix. The multiple linear regression results are presented in S3 Appendix, and the regression coefficients support the findings discussed above.

## Discussion

The first two months of the pandemic in the US (March/April 2020) brought uncertainty and fear as the virus spread. The role of officials’ communication during this period was to bring confidence and transparency to the public to calm their response and enhance their adherence to regulations. Our study found that the numbers of press releases and total words published were significantly higher in the first two months of the pandemic for almost every state. Communication research has observed that frequent communication which mixes formal and informal messages improves communication quality, trust, and collaboration [24]. The frequent communication strategy adopted by states at the early stages of the pandemic corroborates this strategy. Nonetheless, concurrent with observation, researchers in organizational science have shown that trust and trustor predisposition towards the trustee’s mandates decrease if communication frequency is too high [25]. This phenomenon seems to regulate later months in the pandemic, when officials reduced the frequency of press releases and only increased again in reaction to a sudden increase in the numbers of COVID-19 cases and deaths. Our findings agree with the curvilinear model of communication frequency and communication efficacy in organizational theory, which postulates the existence of an optimal communication frequency [26]; our study also supports the utility of extrapolating findings in corporate communication to the less studied government communication field [27]. The results of this study also suggest that officials should rapidly search for an optimal communication frequency with their constituents, thus avoiding the information overload and public uncertainty observed at the beginning of the COVID-19 pandemic.

The larger positivity than negativity observed across states is consistent with findings from corporate communications in crisis situations [28]. In the postulated models, officials try to combat stakeholders’ uncertainty with hopeful and positive messages. Interestingly, we observed that both positive and negative sentiments in press releases were rather homogenous across states at the beginning of the pandemic but then polarized per state as the pandemic evolved. This indicates a stakeholder-based optimal communication tone. Tone optimality has been observed in micro-level communication situations, such as dementia care [29] and patient-doctor quality reports [30]. However, there is no study related to optimal tone for government-level communication in the public health field. The findings of this study suggest the need for officials to rapidly find an optimal communication tone with the public during crisis situations.

Regarding our first research question on how tone evolved over time, we found that both the frequency of press releases and tone were rather uniform at the beginning of the pandemic and then evolved to a state-dependent optimal condition. Our findings suggest that feedback cycles between government officials and public response should be shortened to rapidly yield an optimal communication strategy that maximizes communication efficacy.

The state-dependent optimal conditions in terms of communication tone involve both positive and negative sentiments in the messages transmitted by officials. We expected to find positive sentiments in press releases since they are well-known to mitigate uncertainty and frustration. However, it is interesting that official communications still contained negative sentiments in their messages. Findings in accounting science showed that over-positive messages decrease trustworthiness and credibility in official reports [31]. Conversely, findings in marketing research have observed that two-sided messages, containing both positive and negative tones, may yield a discounted argument importance [32]. Our state-level analyses suggest the discounted importance effect was present since negativity reduced and positivity increased during the pandemic. Nonetheless, remnant negativity was present at each state even after they reached an optimal tone in their communication strategy. This indicates the need to generate trust in official communications via the inclusion of negative sentiments. Moreover, we observed that the level of positivity and negativity in communications across states was markedly different. Hence, addressing our second research question on size of tone fluctuations, we found that optimal communication strategies in the US were state-dependent, mainly positive, and included small amounts of negativity to enhance trustworthiness.

We compared communication tone with the numbers of COVID-19 cases and deaths per state. Our findings showed that official communication was reactive (high correlation with the first time-derivative of the score) to the numbers of COVID-19 cases and deaths, rather than responsive (high correlation with the score) or preventive (high correlation with the second time-derivative of the score). An official reactive response during a crisis can be problematic since it reduces message empathy and strategy, which are key to control uncertainty and discomfort in critical conditions [33]. In terms of tone, micro-level studies have observed that reactivity increases negative predispositions in message recipients, which in turn decreases adherence propensity [34]. Our findings should be expanded by in-depth analysis of the press releases under a sudden increase in the numbers of COVID-19 cases and deaths. Addressing our third research question, we observed the large reactivity in terms of tone in official communication and the reluctant adherence to mandates observed in the US [35], suggesting the need for state officials to better plan public communications during the pandemic.

### Policy recommendations

This is a descriptive study that documents official governor communications during the first nine months of the COVID-19 pandemic. As the global pandemic evolved, the first nine months proved to be the most crucial time period as it included a fair amount of uncertainty in terms of diagnosing the characteristics of the virus, methods of transmission, and understanding its impact. Even with the study’s descriptive nature, we present three key policy recommendations. First, we recommend that health and government offices identify and prepare communication strategies that could be used during large scale events. Second, we suggest these offices review their communication releases during the pandemic to support the overall communication strategy during disaster-like events. Third, while this past pandemic affected regions at different times and magnitude, it is important for the government and state agencies to collaborate and consolidate communication strategies. More research is needed to identify any links between official communication and outcomes.

### Limitations

This study has five limitations. First, the impact of official governors’ press releases on the broad US population is uncertain as the young adults may be frequently unaware of the content of official press releases. Nonetheless, much of the press and alternative information sources (e.g. social media) were based upon press releases, and it is reasonable to expect that the tone set by state officials influenced these communication sources. Second, we excluded official communications by the state health department due to a markedly different tone from that of governors’ offices, leaving these communications for future research. Third, our sentiment analyzer has not been trained using the specific positive and negative lexicon of official press releases; however, we have tailored the architecture of the sentiment analyzer to be more dependent on the sentiment in the message and less dependent on the training context, and we have thoroughly validated its performance by qualitatively analyzing the sentiment assigned to 1,000 randomly selected tokens in official press releases. Fourth, although tone neutrality was predicted by our sentiment analyzer, we have not discussed it here; tone neutrality was more associated with communication risk management during the pandemic and, hence, less indicative of the communication patterns and responsiveness addressed in this study. Finally, our findings must be interpreted with caution as they were just correlations and none of these were statistically significant. While some interesting trends could be observed with relations to political party, age, and gender, there was not enough statistical evidence to univocally associate these factors with the variables of interest. This analysis would guide further research on public health management of crises.

### Conclusion

This study found that official communications in the US reached a state-level optimal equilibrium value in terms of frequency and tone of press releases. However, these equilibrium values differed significantly across states. Official COVID-19 communications were generally positive with only small levels of negativity. Correlations between communication sentiment and the evolution in the numbers of COVID-19 cases and deaths suggest that official COVID-19 communications were reactive to the evolution of the pandemic, rather than responsive or preventive. Future research should investigate the cause of the large differences in communication tone across states and validate the reactive characteristics of COVID-19 official communications.

## Supporting information

S1 Appendix Number of texts, total words, and word density published per state during the study period. (DOCX)

S2 Appendix Sentiment analyzer. (DOCX)

S3 Appendix Multiple linear regression results. (XLSX)

S4 Appendix Scatter plots. (DOCX)
