# Correction: COVID-19 and communication: A sentiment analysis of US state governors’ official press releases

**DOI:** 10.1371/journal.pone.0307783

**Published:** 2024-07-22

**Authors:** Mauricio Tano, Juha Baek, Adriana Ordonez, Rita Bosetti, Terri Menser, George Naufal, Bita Kash

There are errors in the author affiliations. The correct affiliations are as follows:

Mauricio Tano^1^, Juha Baek^2^, Adriana Ordonez^3^, Rita Bosetti^3^, Terri Menser^3,4^, George Naufal^3,5^, Bita Kash^3,6^

**1** Nuclear Engineering Department, Texas A&M University, College Station, TX, USA, **2** Department of Health Care Policy Research, Korea Institute for Health and Social Affairs, Sejong-si 30126, Korea, **3** Center for Outcomes Research, Houston Methodist, Houston, TX, USA, **4** Department of Population Health Sciences, Weill Cornell Medical College, New York, NY, USA, **5** Public Policy Research Institute, Texas A&M University, College Station, TX, USA, **6** Department of Health Policy and Management, School of Public Health, Texas A&M University, College Station, TX, USA.
