# Supplementary material for: COVID-19 and communication: A sentiment analysis of US state governors’ official press releases
Source: PLoS One. 2022 Aug 30;17(8):e0272558. doi: 10.1371/journal.pone.0272558 (PMC9426878; doi:10.1371/journal.pone.0272558)
Supplement: S1 Appendix — (DOCX) [file pone.0272558.s001.docx]

S1 Appendix: Number of texts, total words, and word density published per state during the study period

|  | **Number of Texts** | **Total Words Published** | **Word Density per Text** |
| --- | --- | --- | --- |
| **AK** | 54 | 111184 | 2059 |
| **AL** | 39 | 96169 | 2466 |
| **AR** | 118 | 278664 | 2362 |
| **AZ** | 54 | 150664 | 2790 |
| **CA** | 120 | 417936 | 3483 |
| **CO** | 265 | 753396 | 2843 |
| **CT** | 124 | 490017 | 3952 |
| **DE** | 102 | 388089 | 3805 |
| **FL** | 202 | 3095044 | 15322 |
| **GA** | 111 | 352634 | 3177 |
| **HI** | 285 | 1233165 | 4327 |
| **IA** | 117 | 878118 | 7505 |
| **ID** | 110 | 273622 | 2487 |
| **IL** | 350 | 824974 | 2357 |
| **IN** | 96 | 285661 | 2976 |
| **KS** | 90 | 258999 | 2878 |
| **KY** | 149 | 952865 | 6395 |
| **LA** | 71 | 190444 | 2682 |
| **MA** | 166 | 741194 | 4465 |
| **MD** | 229 | 765680 | 3344 |
| **ME** | 113 | 563112 | 4983 |
| **MI** | 607 | 2216696 | 3652 |
| **MN** | 77 | 198257 | 2575 |
| **MO** | 121 | 427937 | 3537 |
| **MS** | 48 | 45214 | 942 |
| **MT** | 96 | 362877 | 3780 |
| **NC** | 112 | 330168 | 2948 |
| **ND** | 101 | 369919 | 3663 |
| **NE** | 126 | 536613 | 4259 |
| **NH** | 135 | 256341 | 1899 |
| **NJ** | 514 | 11576554 | 22522 |
| **NM** | 75 | 316120 | 4215 |
| **NV** | 74 | 280819 | 3795 |
| **NY** | 90 | 360107 | 4001 |
| **OH** | 103 | 416092 | 4040 |
| **OK** | 35 | 88873 | 2539 |
| **OR** | 117 | 409541 | 3500 |
| **PA** | 135 | 649531 | 4811 |
| **RI** | 70 | 285259 | 4075 |
| **SC** | 126 | 385658 | 3061 |
| **SD** | 58 | 146934 | 2533 |
| **TN** | 111 | 352634 | 3177 |
| **TX** | 181 | 478493 | 2644 |
| **UT** | 218 | 530127 | 2432 |
| **VA** | 194 | 863964 | 4453 |
| **VT** | 103 | 344854 | 3348 |
| **WA** | 218 | 530127 | 2432 |
| **WI** | 187 | 102326 | 547 |
| **WV** | 222 | 148315 | 668 |
| **WY** | 51 | 126354 | 2478 |

**Table. Mean (standard deviation) of negativity per month for all states**

|  | **Mar-20** | **Apr-20** | **May-20** | **Jun-20** | **Jul-20** | **Aug-20** | **Sep-20** | **Oct-20** | **Nov-20** | **Dec-20** |
| --- | --- | --- | --- | --- | --- | --- | --- | --- | --- | --- |
| **AK** | 0.040 (0.004) | 0.021 (0.002) | 0.034 (0.004) | 0.034 (0.002) | 0.000 (0.000) | 0.053 (0.004) | - | 0.055 (0.009) | 0.123 (0.029) | 0.141 (0.009) |
| **AL** | 0.061 (0.006) | 0.029 (0.002) | 0.013 (0.001) | 0.016 (0.001) | 0.026 (0.002) | 0.039 (0.003) | 0.027 (0.002) | 0.014 (0.001) | 0.028 (0.002) | 0.023 (0.002) |
| **AR** | 0.058 (0.007) | 0.020 (0.002) | 0.059 (0.009) | 0.086 (0.013) | 0.036 (0.004) | 0.046 (0.006) | 0.021 (0.002) | 0.023 (0.003) | 0.019 (0.002) | 0.022 (0.002) |
| **AZ** | - | 0.037 (0.003) | 0.026 (0.001) | 0.033 (0.001) | 0.028 (0.003) | 0.036 (0.004) | 0.037 (0.003) | 0.041 (0.004) | 0.021 (0.001) | 0.038 (0.004) |
| **CA** | 0.056 (0.006) | 0.047 (0.004) | 0.052 (0.006) | 0.027 (0.002) | 0.034 (0.003) | 0.039 (0.004) | 0.036 (0.003) | 0.021 (0.002) | 0.043 (0.004) | 0.035 (0.003) |
| **CO** | 0.038 (0.004) | 0.028 (0.003) | 0.040 (0.006) | 0.023 (0.002) | 0.032 (0.004) | 0.030 (0.003) | 0.027 (0.003) | 0.023 (0.002) | 0.031 (0.003) | 0.034 (0.004) |
| **CT** | 0.058 (0.008) | 0.042 (0.004) | 0.030 (0.002) | 0.041 (0.004) | 0.030 (0.004) | 0.043 (0.004) | 0.045 (0.004) | 0.014 (0.001) | 0.041 (0.003) | 0.037 (0.004) |
| **DE** | 0.047 (0.006) | 0.055 (0.011) | 0.033 (0.005) | 0.029 (0.003) | 0.045 (0.007) | 0.051 (0.008) | 0.039 (0.007) | 0.055 (0.011) | 0.032 (0.005) | 0.043 (0.007) |
| **FL** | 0.039 (0.005) | 0.021 (0.002) | 0.026 (0.003) | 0.025 (0.003) | 0.017 (0.002) | 0.017 (0.002) | 0.023 (0.003) | 0.014 (0.002) | 0.011 (0.002) | 0.004 (0.000) |
| **GA** | 0.067 (0.009) | 0.028 (0.002) | 0.032 (0.002) | 0.047 (0.005) | 0.029 (0.002) | 0.070 (0.009) | 0.056 (0.005) | 0.050 (0.005) | 0.028 (0.002) | 0.018 (0.001) |
| **HI** | 0.053 (0.008) | 0.063 (0.007) | 0.047 (0.007) | 0.047 (0.006) | 0.043 (0.005) | 0.048 (0.007) | 0.046 (0.007) | 0.033 (0.005) | 0.046 (0.007) | 0.047 (0.007) |
| **IA** | 0.041 (0.006) | 0.040 (0.005) | - | 0.022 (0.002) | 0.010 (0.001) | 0.026 (0.003) | 0.026 (0.002) | 0.026 (0.002) | 0.035 (0.004) | 0.033 (0.003) |
| **ID** | 0.054 (0.007) | 0.031 (0.003) | 0.024 (0.002) | 0.025 (0.003) | 0.019 (0.001) | 0.014 (0.001) | 0.030 (0.003) | 0.025 (0.002) | 0.092 (0.011) | 0.040 (0.006) |
| **IL** | 0.029 (0.003) | 0.006 (0.000) | 0.007 (0.000) | 0.028 (0.005) | 0.022 (0.004) | 0.029 (0.005) | 0.034 (0.006) | 0.027 (0.004) | 0.006 (0.000) | 0.015 (0.002) |
| **IN** | 0.043 (0.006) | 0.033 (0.003) | 0.018 (0.001) | 0.031 (0.004) | 0.019 (0.002) | 0.026 (0.003) | 0.003 (0.000) | 0.026 (0.004) | - | 0.029 (0.002) |
| **KS** | 0.055 (0.006) | 0.056 (0.006) | 0.067 (0.010) | 0.032 (0.003) | 0.052 (0.008) | 0.045 (0.003) | 0.068 (0.008) | 0.037 (0.003) | 0.033 (0.002) | 0.036 (0.003) |
| **KY** | 0.037 (0.006) | 0.033 (0.007) | 0.040 (0.010) | 0.012 (0.001) | 0.039 (0.007) | 0.028 (0.003) | 0.067 (0.012) | 0.040 (0.007) | 0.044 (0.007) | 0.029 (0.003) |
| **LA** | 0.049 (0.006) | 0.032 (0.005) | 0.032 (0.005) | 0.042 (0.005) | 0.042 (0.004) | 0.036 (0.003) | - | 0.041 (0.004) | 0.014 (0.001) | - |
| **MA** | 0.044 (0.005) | 0.033 (0.003) | 0.028 (0.003) | 0.024 (0.002) | 0.030 (0.003) | 0.031 (0.003) | 0.035 (0.003) | 0.033 (0.004) | 0.027 (0.002) | 0.028 (0.003) |
| **MD** | 0.053 (0.006) | 0.044 (0.006) | 0.024 (0.003) | 0.038 (0.005) | 0.040 (0.005) | 0.038 (0.004) | 0.043 (0.005) | 0.030 (0.003) | 0.041 (0.005) | 0.036 (0.004) |
| **ME** | 0.040 (0.004) | 0.039 (0.003) | 0.032 (0.003) | 0.036 (0.004) | 0.022 (0.002) | 0.033 (0.003) | 0.044 (0.004) | 0.054 (0.005) | 0.034 (0.004) | 0.035 (0.003) |
| **MI** | 0.046 (0.007) | 0.042 (0.005) | 0.033 (0.004) | 0.032 (0.004) | 0.028 (0.003) | 0.034 (0.003) | 0.029 (0.003) | 0.039 (0.005) | 0.029 (0.003) | 0.035 (0.004) |
| **MN** | 0.051 (0.005) | 0.033 (0.002) | 0.033 (0.002) | 0.037 (0.004) | 0.047 (0.006) | 0.046 (0.004) | 0.051 (0.006) | 0.036 (0.004) | 0.037 (0.006) | 0.031 (0.003) |
| **MO** | 0.050 (0.006) | 0.035 (0.004) | 0.051 (0.008) | 0.045 (0.006) | 0.042 (0.007) | 0.055 (0.007) | 0.026 (0.002) | 0.029 (0.003) | 0.043 (0.005) | 0.015 (0.001) |
| **MS** | - | - | 0.045 (0.006) | 0.079 (0.017) | 0.082 (0.018) | 0.047 (0.008) | 0.039 (0.004) | - | - | - |
| **MT** | 0.050 (0.007) | 0.043 (0.004) | 0.022 (0.002) | 0.034 (0.003) | 0.046 (0.007) | 0.041 (0.004) | 0.045 (0.005) | 0.031 (0.003) | 0.026 (0.002) | 0.057 (0.009) |
| **NC** | 0.062 (0.008) | 0.034 (0.003) | 0.028 (0.002) | 0.017 (0.001) | 0.030 (0.004) | 0.034 (0.003) | 0.033 (0.003) | 0.043 (0.006) | 0.033 (0.004) | 0.030 (0.004) |
| **ND** | 0.044 (0.005) | 0.049 (0.006) | 0.034 (0.004) | 0.028 (0.004) | 0.031 (0.004) | 0.055 (0.006) | 0.049 (0.005) | 0.031 (0.003) | 0.035 (0.003) | 0.021 (0.002) |
| **NE** | 0.031 (0.004) | 0.045 (0.011) | 0.030 (0.005) | 0.035 (0.004) | 0.016 (0.002) | 0.016 (0.002) | 0.031 (0.003) | 0.018 (0.002) | 0.021 (0.003) | 0.021 (0.003) |
| **NH** | 0.039 (0.003) | 0.065 (0.008) | 0.037 (0.003) | 0.007 (0.000) | 0.038 (0.005) | 0.055 (0.009) | 0.034 (0.004) | 0.050 (0.010) | 0.036 (0.005) | - |
| **NJ** | 0.041 (0.005) | 0.055 (0.008) | 0.050 (0.007) | 0.034 (0.005) | 0.039 (0.005) | 0.047 (0.006) | 0.029 (0.004) | 0.043 (0.005) | 0.045 (0.006) | 0.036 (0.005) |
| **NM** | 0.052 (0.006) | 0.056 (0.008) | 0.042 (0.004) | 0.027 (0.002) | 0.048 (0.006) | 0.041 (0.003) | 0.037 (0.003) | 0.066 (0.009) | 0.056 (0.009) | - |
| **NV** | 0.034 (0.003) | 0.044 (0.004) | 0.025 (0.003) | 0.020 (0.002) | 0.028 (0.002) | 0.058 (0.006) | 0.008 (0.000) | 0.017 (0.002) | 0.025 (0.003) | 0.022 (0.002) |
| **NY** | 0.040 (0.004) | 0.033 (0.003) | 0.057 (0.005) | 0.044 (0.007) | 0.049 (0.005) | - | 0.046 (0.005) | 0.036 (0.004) | 0.040 (0.005) | 0.016 (0.001) |
| **OH** | 0.052 (0.007) | 0.039 (0.006) | 0.019 (0.002) | 0.030 (0.006) | 0.025 (0.003) | 0.043 (0.007) | 0.022 (0.002) | 0.036 (0.005) | 0.033 (0.004) | 0.021 (0.002) |
| **OK** | 0.050 (0.005) | 0.028 (0.002) | - | 0.009 (0.000) | 0.032 (0.003) | 0.026 (0.002) | 0.011 (0.001) | 0.032 (0.002) | 0.035 (0.002) | 0.027 (0.002) |
| **OR** | 0.053 (0.006) | 0.060 (0.008) | 0.027 (0.003) | 0.029 (0.003) | 0.026 (0.003) | 0.034 (0.004) | 0.049 (0.007) | 0.028 (0.003) | 0.040 (0.004) | 0.045 (0.006) |
| **PA** | 0.044 (0.005) | 0.057 (0.007) | 0.020 (0.002) | 0.037 (0.003) | 0.039 (0.004) | 0.032 (0.003) | 0.032 (0.003) | 0.035 (0.004) | 0.040 (0.004) | 0.046 (0.005) |
| **RI** | 0.055 (0.008) | 0.066 (0.010) | 0.066 (0.010) | 0.058 (0.010) | 0.028 (0.003) | 0.036 (0.005) | 0.043 (0.006) | 0.003 (0.000) | 0.023 (0.003) | 0.014 (0.002) |
| **SC** | 0.044 (0.006) | 0.041 (0.006) | 0.032 (0.005) | 0.067 (0.011) | 0.022 (0.003) | 0.029 (0.004) | 0.031 (0.004) | 0.024 (0.004) | 0.046 (0.006) | 0.054 (0.008) |
| **SD** | - | - | 0.065 (0.020) | 0.051 (0.009) | 0.047 (0.006) | 0.068 (0.019) | 0.042 (0.006) | 0.033 (0.005) | 0.016 (0.001) | 0.015 (0.001) |
| **TN** | 0.067 (0.009) | 0.028 (0.002) | 0.032 (0.002) | 0.047 (0.005) | 0.029 (0.002) | 0.070 (0.009) | 0.056 (0.005) | 0.050 (0.005) | 0.028 (0.002) | 0.018 (0.001) |
| **TX** | 0.052 (0.006) | 0.039 (0.004) | 0.032 (0.003) | 0.030 (0.003) | 0.033 (0.003) | 0.021 (0.002) | 0.055 (0.006) | 0.040 (0.005) | 0.045 (0.004) | 0.035 (0.004) |
| **UT** | 0.061 (0.010) | 0.066 (0.010) | 0.032 (0.003) | 0.040 (0.004) | 0.041 (0.005) | 0.049 (0.006) | 0.076 (0.007) | 0.052 (0.007) | 0.037 (0.004) | 0.031 (0.004) |
| **VA** | 0.051 (0.007) | 0.036 (0.004) | 0.040 (0.005) | 0.026 (0.003) | 0.030 (0.003) | 0.044 (0.005) | 0.031 (0.003) | 0.031 (0.003) | 0.031 (0.003) | 0.030 (0.003) |
| **VT** | 0.048 (0.006) | 0.038 (0.004) | 0.030 (0.003) | 0.043 (0.004) | 0.026 (0.002) | 0.026 (0.002) | 0.036 (0.005) | 0.036 (0.003) | 0.052 (0.006) | 0.038 (0.004) |
| **WA** | 0.061 (0.010) | 0.066 (0.010) | 0.032 (0.003) | 0.040 (0.004) | 0.041 (0.005) | 0.049 (0.006) | 0.076 (0.007) | 0.052 (0.007) | 0.037 (0.004) | 0.031 (0.004) |
| **WI** | 0.000 (0.000) | 0.000 (0.000) | 0.000 (0.000) | 0.000 (0.000) | 0.000 (0.000) | 0.000 (0.000) | - | 0.000 (0.000) | 0.001 (0.000) | 0.002 (0.000) |
| **WV** | 0.012 (0.001) | 0.022 (0.003) | 0.005 (0.000) | 0.010 (0.001) | 0.001 (0.000) | 0.007 (0.000) | 0.017 (0.001) | 0.015 (0.001) | 0.009 (0.001) | 0.002 (0.000) |
| **WY** | 0.033 (0.004) | 0.027 (0.003) | 0.027 (0.004) | 0.037 (0.006) | 0.036 (0.004) | 0.024 (0.003) | 0.032 (0.004) | 0.028 (0.003) | 0.035 (0.004) | 0.030 (0.003) |

**Table. Mean (standard deviation) of the positivity per month for all states**

|  | **Mar-20** | **Apr-20** | **May-20** | **Jun-20** | **Jul-20** | **Aug-20** | **Sep-20** | **Oct-20** | **Nov-20** | **Dec-20** |
| --- | --- | --- | --- | --- | --- | --- | --- | --- | --- | --- |
| **AK** | 0.078 (0.007) | 0.092 (0.008) | 0.103 (0.012) | 0.070 (0.005) | 0.078 (0.008) | 0.053 (0.007) | - | 0.086 (0.010) | 0.087 (0.014) | 0.022 (0.001) |
| **AL** | 0.061 (0.009) | 0.132 (0.011) | 0.133 (0.004) | 0.121 (0.010) | 0.131 (0.010) | 0.132 (0.009) | 0.152 (0.009) | 0.155 (0.017) | 0.132 (0.009) | 0.138 (0.008) |
| **AR** | 0.073 (0.008) | 0.089 (0.010) | 0.071 (0.011) | 0.091 (0.011) | 0.085 (0.014) | 0.080 (0.012) | 0.067 (0.006) | 0.064 (0.007) | 0.068 (0.006) | 0.068 (0.009) |
| **AZ** | - | 0.138 (0.009) | 0.131 (0.012) | 0.071 (0.004) | 0.118 (0.013) | 0.107 (0.015) | 0.111 (0.007) | 0.207 (0.009) | 0.133 (0.014) | 0.141 (0.014) |
| **CA** | 0.109 (0.011) | 0.114 (0.010) | 0.111 (0.010) | 0.092 (0.008) | 0.112 (0.010) | 0.088 (0.008) | 0.119 (0.011) | 0.091 (0.009) | 0.118 (0.011) | 0.112 (0.011) |
| **CO** | 0.075 (0.009) | 0.076 (0.010) | 0.096 (0.012) | 0.110 (0.012) | 0.097 (0.011) | 0.092 (0.008) | 0.102 (0.010) | 0.085 (0.008) | 0.077 (0.010) | 0.090 (0.010) |
| **CT** | 0.090 (0.009) | 0.107 (0.010) | 0.093 (0.009) | 0.112 (0.011) | 0.119 (0.009) | 0.125 (0.009) | 0.096 (0.009) | 0.117 (0.010) | 0.109 (0.011) | 0.101 (0.007) |
| **DE** | 0.059 (0.008) | 0.080 (0.012) | 0.070 (0.011) | 0.079 (0.012) | 0.085 (0.013) | 0.063 (0.008) | 0.065 (0.009) | 0.077 (0.013) | 0.064 (0.012) | 0.081 (0.012) |
| **FL** | 0.090 (0.009) | 0.072 (0.009) | 0.078 (0.012) | 0.087 (0.015) | 0.092 (0.014) | 0.115 (0.013) | 0.133 (0.015) | 0.135 (0.016) | 0.131 (0.017) | 0.084 (0.012) |
| **GA** | 0.079 (0.009) | 0.092 (0.011) | 0.112 (0.009) | 0.127 (0.011) | 0.145 (0.020) | 0.104 (0.016) | 0.083 (0.008) | 0.086 (0.009) | 0.079 (0.011) | 0.085 (0.009) |
| **HI** | 0.084 (0.010) | 0.083 (0.008) | 0.088 (0.010) | 0.070 (0.007) | 0.067 (0.008) | 0.063 (0.008) | 0.077 (0.009) | 0.078 (0.009) | 0.078 (0.010) | 0.107 (0.015) |
| **IA** | 0.051 (0.006) | 0.066 (0.007) | - | 0.079 (0.008) | 0.084 (0.009) | 0.087 (0.011) | 0.098 (0.010) | 0.097 (0.012) | 0.078 (0.008) | 0.118 (0.010) |
| **ID** | 0.085 (0.010) | 0.097 (0.011) | 0.096 (0.011) | 0.102 (0.010) | 0.119 (0.010) | 0.132 (0.017) | 0.135 (0.013) | 0.102 (0.013) | 0.160 (0.019) | 0.124 (0.017) |
| **IL** | 0.045 (0.004) | 0.006 (0.000) | 0.025 (0.003) | 0.032 (0.004) | 0.035 (0.004) | 0.043 (0.005) | 0.038 (0.005) | 0.041 (0.006) | 0.056 (0.008) | 0.034 (0.004) |
| **IN** | 0.061 (0.006) | 0.069 (0.007) | 0.077 (0.009) | 0.094 (0.011) | 0.087 (0.010) | 0.105 (0.011) | 0.056 (0.008) | 0.078 (0.014) | - | 0.070 (0.006) |
| **KS** | 0.099 (0.009) | 0.097 (0.010) | 0.099 (0.011) | 0.144 (0.013) | 0.117 (0.007) | 0.151 (0.014) | 0.119 (0.010) | 0.119 (0.012) | 0.154 (0.011) | 0.105 (0.010) |
| **KY** | 0.075 (0.011) | 0.074 (0.011) | 0.076 (0.012) | 0.077 (0.009) | 0.091 (0.015) | 0.099 (0.012) | 0.078 (0.012) | 0.074 (0.010) | 0.067 (0.009) | 0.091 (0.013) |
| **LA** | 0.066 (0.009) | 0.073 (0.010) | 0.081 (0.009) | 0.073 (0.006) | 0.075 (0.011) | 0.074 (0.008) | - | 0.060 (0.006) | 0.062 (0.007) | - |
| **MA** | 0.075 (0.008) | 0.090 (0.009) | 0.090 (0.008) | 0.085 (0.008) | 0.091 (0.008) | 0.098 (0.010) | 0.092 (0.008) | 0.113 (0.011) | 0.093 (0.011) | 0.103 (0.013) |
| **MD** | 0.072 (0.007) | 0.096 (0.012) | 0.084 (0.009) | 0.084 (0.010) | 0.078 (0.010) | 0.119 (0.012) | 0.087 (0.010) | 0.117 (0.015) | 0.105 (0.011) | 0.085 (0.010) |
| **ME** | 0.113 (0.011) | 0.112 (0.010) | 0.103 (0.008) | 0.106 (0.010) | 0.133 (0.013) | 0.100 (0.010) | 0.088 (0.008) | 0.077 (0.008) | 0.126 (0.013) | 0.128 (0.013) |
| **MI** | 0.065 (0.008) | 0.080 (0.009) | 0.090 (0.011) | 0.093 (0.010) | 0.094 (0.010) | 0.078 (0.009) | 0.083 (0.010) | 0.080 (0.009) | 0.087 (0.011) | 0.072 (0.008) |
| **MN** | 0.094 (0.009) | 0.177 (0.013) | 0.165 (0.012) | 0.145 (0.016) | 0.104 (0.010) | 0.083 (0.004) | 0.103 (0.009) | 0.095 (0.008) | 0.096 (0.013) | 0.091 (0.012) |
| **MO** | 0.085 (0.009) | 0.106 (0.010) | 0.106 (0.013) | 0.077 (0.011) | 0.125 (0.012) | 0.121 (0.012) | 0.132 (0.013) | 0.154 (0.015) | 0.089 (0.007) | 0.143 (0.011) |
| **MS** | - | - | 0.071 (0.005) | 0.103 (0.012) | 0.061 (0.009) | 0.064 (0.010) | 0.089 (0.008) | - | - | - |
| **MT** | 0.084 (0.009) | 0.101 (0.010) | 0.103 (0.010) | 0.111 (0.012) | 0.111 (0.012) | 0.114 (0.012) | 0.108 (0.013) | 0.082 (0.008) | 0.106 (0.011) | 0.076 (0.007) |
| **NC** | 0.094 (0.008) | 0.098 (0.009) | 0.099 (0.010) | 0.106 (0.014) | 0.084 (0.009) | 0.102 (0.013) | 0.118 (0.011) | 0.117 (0.014) | 0.086 (0.010) | 0.092 (0.011) |
| **ND** | 0.080 (0.009) | 0.088 (0.009) | 0.101 (0.010) | 0.120 (0.011) | 0.116 (0.010) | 0.099 (0.009) | 0.136 (0.015) | 0.117 (0.011) | 0.151 (0.013) | 0.100 (0.011) |
| **NE** | 0.062 (0.008) | 0.088 (0.014) | 0.094 (0.016) | 0.121 (0.017) | 0.094 (0.012) | 0.097 (0.013) | 0.086 (0.012) | 0.120 (0.013) | 0.112 (0.018) | 0.100 (0.018) |
| **NH** | 0.077 (0.007) | 0.083 (0.008) | 0.079 (0.006) | 0.219 (0.012) | 0.074 (0.011) | 0.079 (0.013) | 0.083 (0.011) | 0.074 (0.015) | 0.103 (0.012) | - |
| **NJ** | 0.091 (0.011) | 0.099 (0.013) | 0.092 (0.012) | 0.117 (0.019) | 0.104 (0.015) | 0.099 (0.012) | 0.111 (0.016) | 0.102 (0.014) | 0.092 (0.015) | 0.112 (0.016) |
| **NM** | 0.081 (0.009) | 0.072 (0.007) | 0.080 (0.011) | 0.106 (0.007) | 0.090 (0.009) | 0.089 (0.009) | 0.110 (0.015) | 0.093 (0.014) | 0.057 (0.006) | - |
| **NV** | 0.091 (0.006) | 0.093 (0.011) | 0.060 (0.007) | 0.094 (0.009) | 0.086 (0.009) | 0.057 (0.006) | 0.092 (0.012) | 0.056 (0.007) | 0.057 (0.007) | 0.088 (0.013) |
| **NY** | 0.092 (0.007) | 0.086 (0.008) | 0.078 (0.005) | 0.105 (0.006) | 0.077 (0.009) | - | 0.059 (0.003) | 0.087 (0.008) | 0.121 (0.011) | 0.058 (0.004) |
| **OH** | 0.071 (0.009) | 0.075 (0.010) | 0.087 (0.011) | 0.081 (0.010) | 0.083 (0.009) | 0.064 (0.010) | 0.080 (0.009) | 0.072 (0.008) | 0.073 (0.010) | 0.068 (0.009) |
| **OK** | 0.098 (0.008) | 0.087 (0.010) | - | 0.112 (0.005) | 0.090 (0.009) | 0.073 (0.007) | 0.073 (0.009) | 0.034 (0.003) | 0.111 (0.011) | 0.092 (0.008) |
| **OR** | 0.099 (0.010) | 0.124 (0.011) | 0.116 (0.012) | 0.098 (0.011) | 0.089 (0.009) | 0.073 (0.007) | 0.074 (0.010) | 0.101 (0.015) | 0.116 (0.019) | 0.105 (0.013) |
| **PA** | 0.089 (0.009) | 0.103 (0.011) | 0.105 (0.008) | 0.081 (0.008) | 0.080 (0.009) | 0.097 (0.009) | 0.099 (0.010) | 0.085 (0.009) | 0.080 (0.008) | 0.098 (0.011) |
| **RI** | 0.069 (0.013) | 0.046 (0.007) | 0.048 (0.008) | 0.051 (0.008) | 0.085 (0.010) | 0.052 (0.006) | 0.050 (0.004) | 0.201 (0.015) | 0.118 (0.017) | 0.065 (0.008) |
| **SC** | 0.070 (0.007) | 0.068 (0.008) | 0.083 (0.010) | 0.109 (0.009) | 0.130 (0.015) | 0.077 (0.011) | 0.049 (0.005) | 0.077 (0.009) | 0.116 (0.013) | 0.081 (0.009) |
| **SD** | - | - | 0.082 (0.012) | 0.114 (0.013) | 0.064 (0.008) | 0.083 (0.014) | 0.118 (0.013) | 0.097 (0.014) | 0.045 (0.009) | 0.077 (0.009) |
| **TN** | 0.079 (0.009) | 0.092 (0.011) | 0.112 (0.009) | 0.127 (0.011) | 0.145 (0.020) | 0.104 (0.016) | 0.083 (0.008) | 0.086 (0.009) | 0.079 (0.011) | 0.085 (0.009) |
| **TX** | 0.102 (0.009) | 0.103 (0.012) | 0.078 (0.007) | 0.094 (0.011) | 0.106 (0.010) | 0.094 (0.008) | 0.085 (0.014) | 0.071 (0.005) | 0.092 (0.008) | 0.080 (0.008) |
| **UT** | 0.074 (0.010) | 0.062 (0.007) | 0.100 (0.010) | 0.048 (0.007) | 0.077 (0.009) | 0.057 (0.007) | 0.046 (0.006) | 0.102 (0.013) | 0.060 (0.007) | 0.107 (0.015) |
| **VA** | 0.076 (0.008) | 0.099 (0.008) | 0.100 (0.010) | 0.113 (0.011) | 0.125 (0.012) | 0.126 (0.010) | 0.099 (0.010) | 0.133 (0.012) | 0.102 (0.008) | 0.118 (0.010) |
| **VT** | 0.093 (0.010) | 0.098 (0.009) | 0.114 (0.013) | 0.090 (0.009) | 0.102 (0.009) | 0.102 (0.009) | 0.065 (0.008) | 0.097 (0.009) | 0.103 (0.011) | 0.108 (0.009) |
| **WA** | 0.074 (0.010) | 0.062 (0.007) | 0.100 (0.010) | 0.048 (0.007) | 0.077 (0.009) | 0.057 (0.007) | 0.046 (0.006) | 0.102 (0.013) | 0.060 (0.007) | 0.107 (0.015) |
| **WI** | 0.110 (0.005) | 0.110 (0.005) | 0.112 (0.005) | 0.113 (0.005) | 0.113 (0.005) | 0.110 (0.005) | - | 0.109 (0.005) | 0.112 (0.005) | 0.108 (0.005) |
| **WV** | 0.141 (0.022) | 0.142 (0.015) | 0.101 (0.005) | 0.176 (0.021) | 0.183 (0.025) | 0.148 (0.015) | 0.148 (0.014) | 0.184 (0.019) | 0.190 (0.021) | 0.138 (0.014) |
| **WY** | 0.082 (0.010) | 0.069 (0.007) | 0.113 (0.011) | 0.115 (0.009) | 0.091 (0.009) | 0.096 (0.008) | 0.096 (0.008) | 0.103 (0.011) | 0.097 (0.011) | 0.118 (0.011) |
