# Supplementary material for: COVID-19 and communication: A sentiment analysis of US state governors’ official press releases
Source: PLoS One. 2022 Aug 30;17(8):e0272558. doi: 10.1371/journal.pone.0272558 (PMC9426878; doi:10.1371/journal.pone.0272558)
Supplement: S2 Appendix — (DOCX) [file pone.0272558.s002.docx]

**S2 Appendix**

**Sentiment analyzer architecture**

A schematic diagram of the analyzer can be found in Figure A1. First, the co-occurrence matrix for text segments (tokens) is computed. The co-occurrence matrix is encoded by means of two parallel convolutional layers of 25 channels of 5x5 convolutional filters using reflection padding and stride=1. The architecture of the network is then divided in two: (i) an attention mechanism (left branch in Figure A1) and (ii) a compression mechanism (right branch in Figure A1). In the attention mechanism, the output of each convolutional filter is passed through a 5x5 max-pooling operation with stride of 4 (compression factor of 4), added and flattened in a merge layer, and passed through an attention layer to yield the attention vector. The output attention vector has 100 components. In the compression mechanism, the output of the right convolutional filter is flattened and compressed in a fully connected encoder to 100 components. Missing entries in the input to the encoder are completed with zeros. Finally, the output of the encoder is activated (multiplied) with the activation vector and fed into a sentic LSTM network with architecture and outputs equivalent to the one proposed by Ma et al.^1^ The resulting network has ~13 million free parameters and was trained until early stopped by overfitting for ~1,200 epochs. The models were trained on the following standard dictionaries:

- VADER,^2^
- Paper Reviews Data Set,^3^
- Standford’s sentiment treebank,^4^
- Sentiment 140,^5^
- OpenRank Reviews.^6^


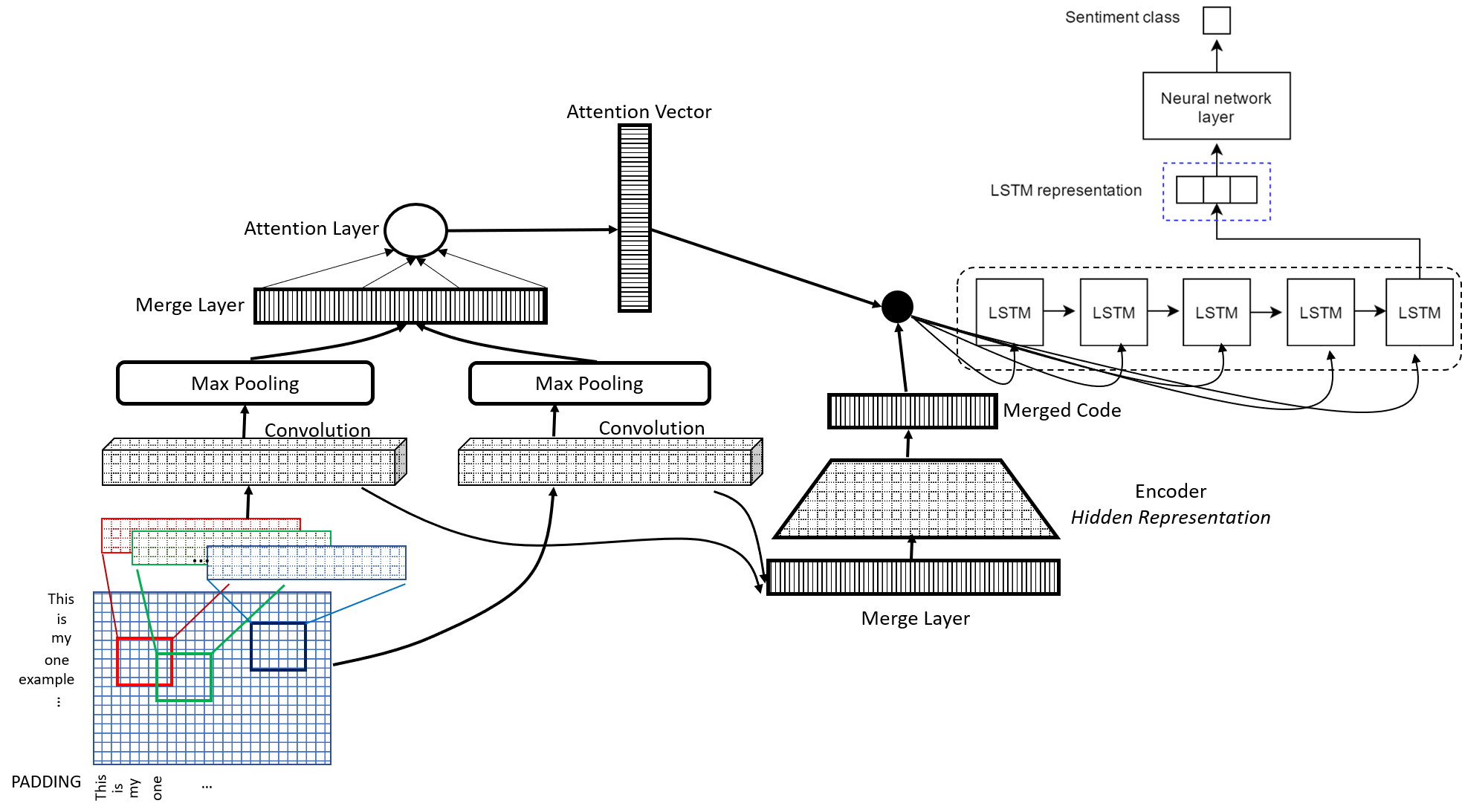


**Figure A1. Architecture of the sentiment analyzer designed for this work**

**References for supplemental materials**

1. Ma Y, Peng H, Khan T, Cambria E, Hussain A. Sentic LSTM: a hybrid network for targeted aspect-based sentiment analysis. Cognit Comput. 2018;10:639–650.

2. Hutto C, Gilbert E. VADER: a parsimonious rule-based model for sentiment analysis of social media text. Proceedings of the International AAAI Conference on Web and Social Media. 2014;8:216–225. Available from: https://ojs.aaai.org/index.php/ICWSM/article/view/14550

3. Keith B, Fuentes E, Meneses C. A hybrid approach for sentiment analysis applied to paper. In: Proceedings of ACM SIGKDD Conference; 2017 August; Halifax, Nova Scotia, Canada; 2017. p. 10. Available from: https://sentic.net/wisdom2017fuentes.pdf

4. Socher R, Perelygin A, Wu J, Chuang J, Manning CD, Ng AY, Potts C. Recursive deep models for semantic compositionality over a sentiment treebank. In: Proceedings of the 2013 Conference on Empirical Methods in Natural Language Processing; Seattle, WA: Association for Computational Linguistics; 2013, pp. 1631–1642.

5. http://help.sentiment140.com [Internet]. Sentiment140 [cited 2021 July 1]. Available at: http://help.sentiment140.com/api

6. Qureshi MS, Daud A, Hayat MK, Afzal MT. OpenRank--a novel approach to rank universities using objective and publicly verifiable data sources. Library Hi Tech. 2021 Jan 1. doi: 10.1108/LHT-07-2019-0131.
