# Supplementary material for: COVID-19 and communication: A sentiment analysis of US state governors’ official press releases
Source: PLoS One. 2022 Aug 30;17(8):e0272558. doi: 10.1371/journal.pone.0272558 (PMC9426878; doi:10.1371/journal.pone.0272558)

S4 Appendix: Scatter Plots

Total articles released per month per state


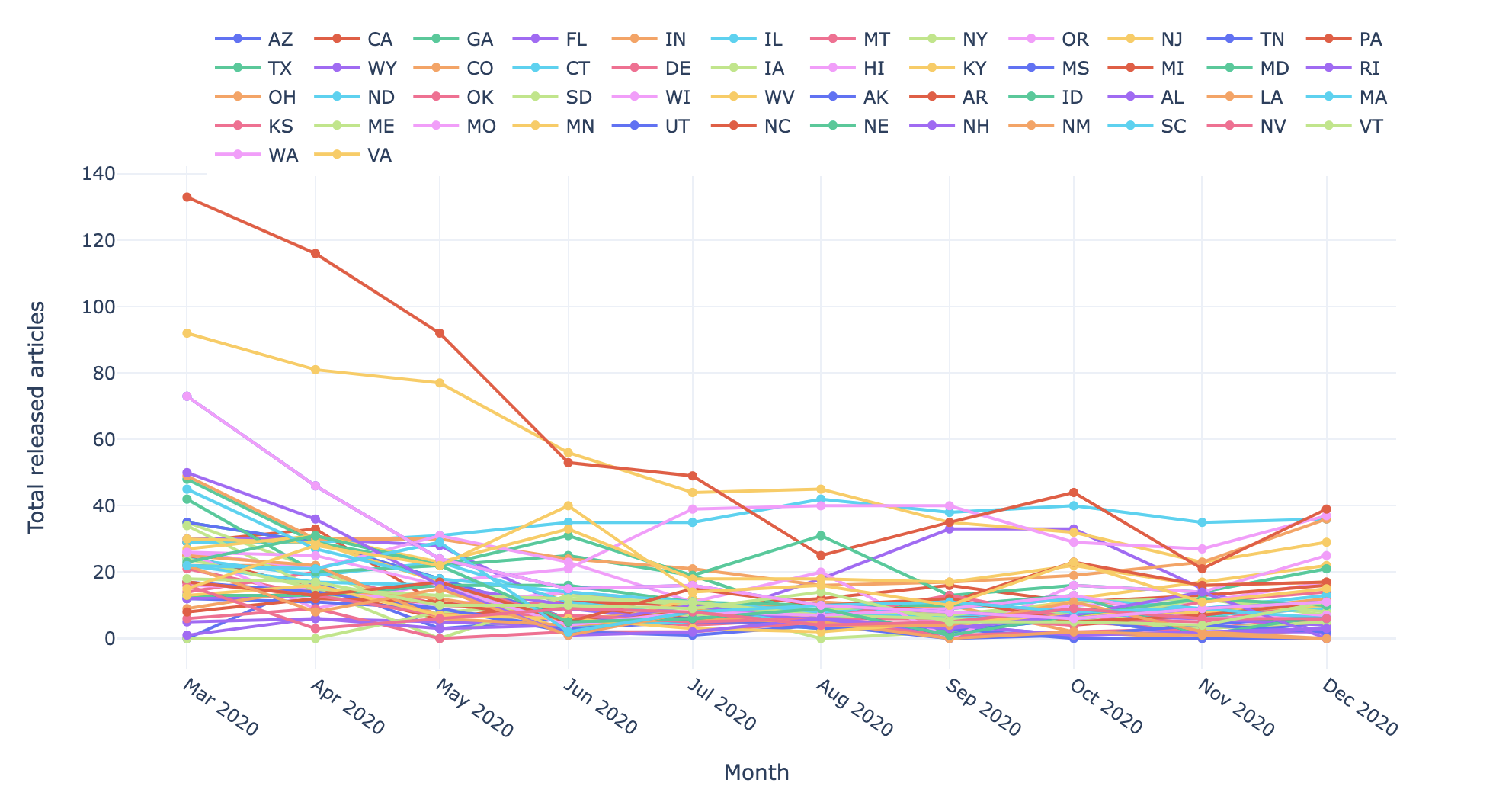


Distribution of negativity in press releases (in absolute value) per month and per state


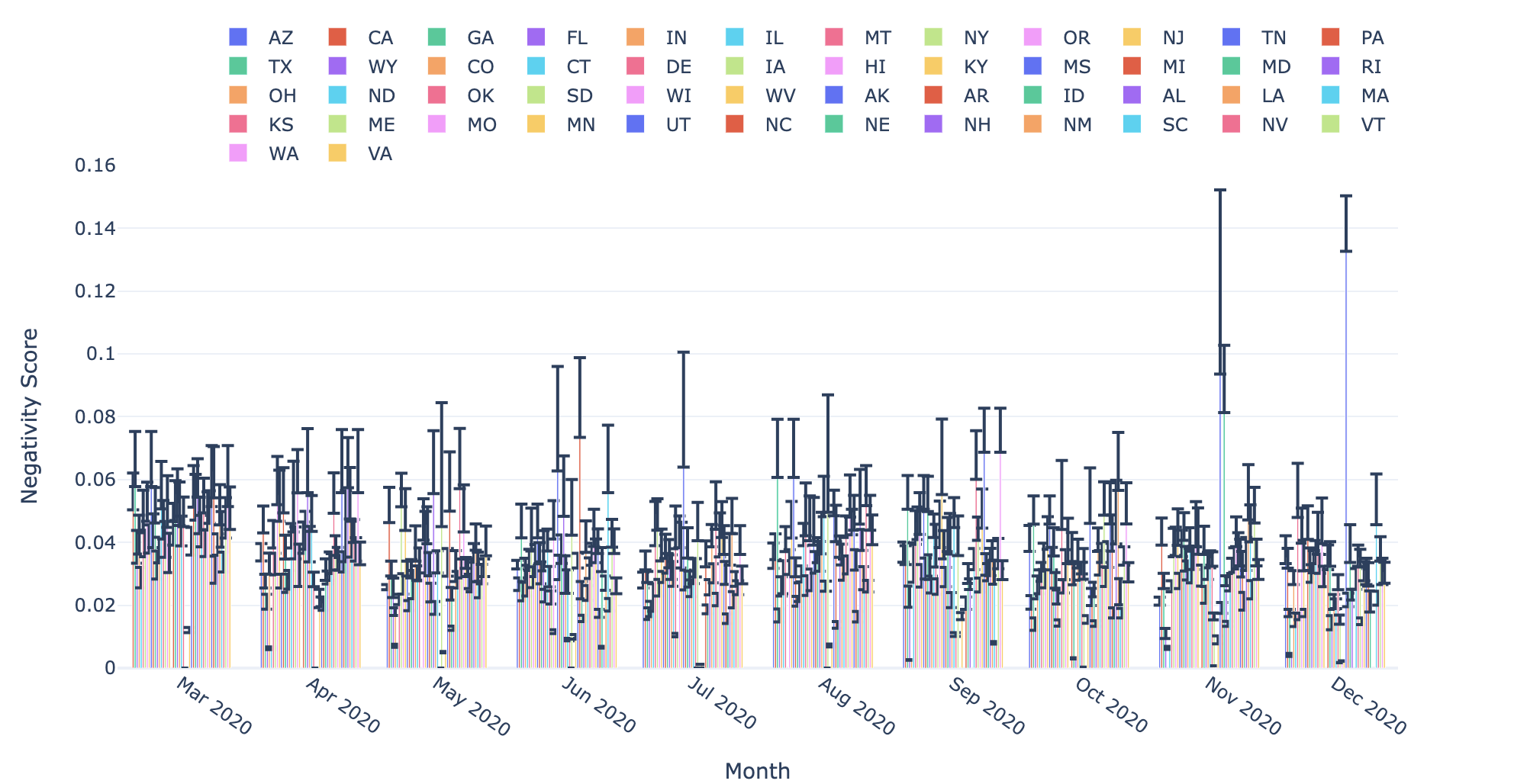


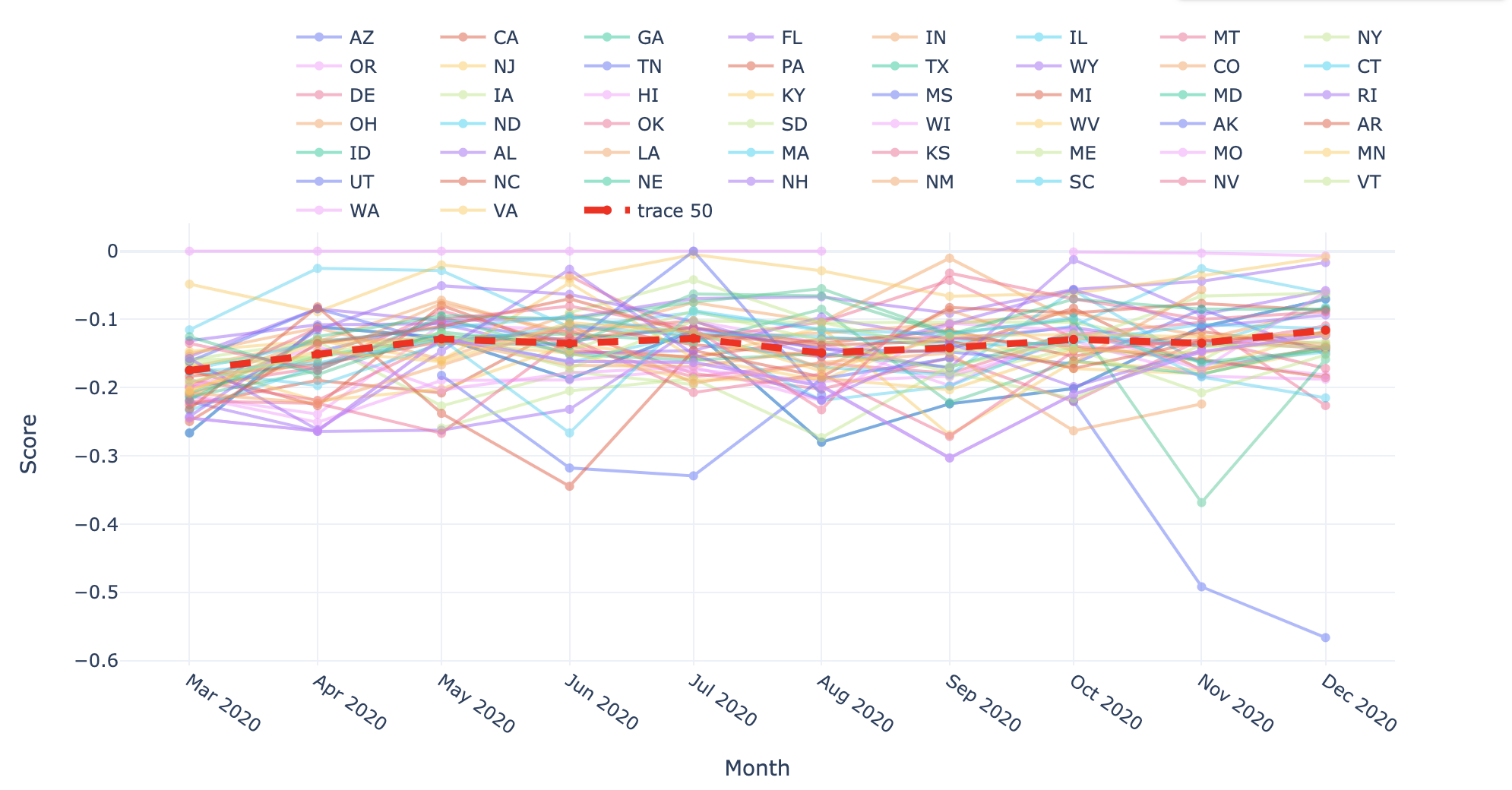


Distribution of positivity in press releases per month and per state


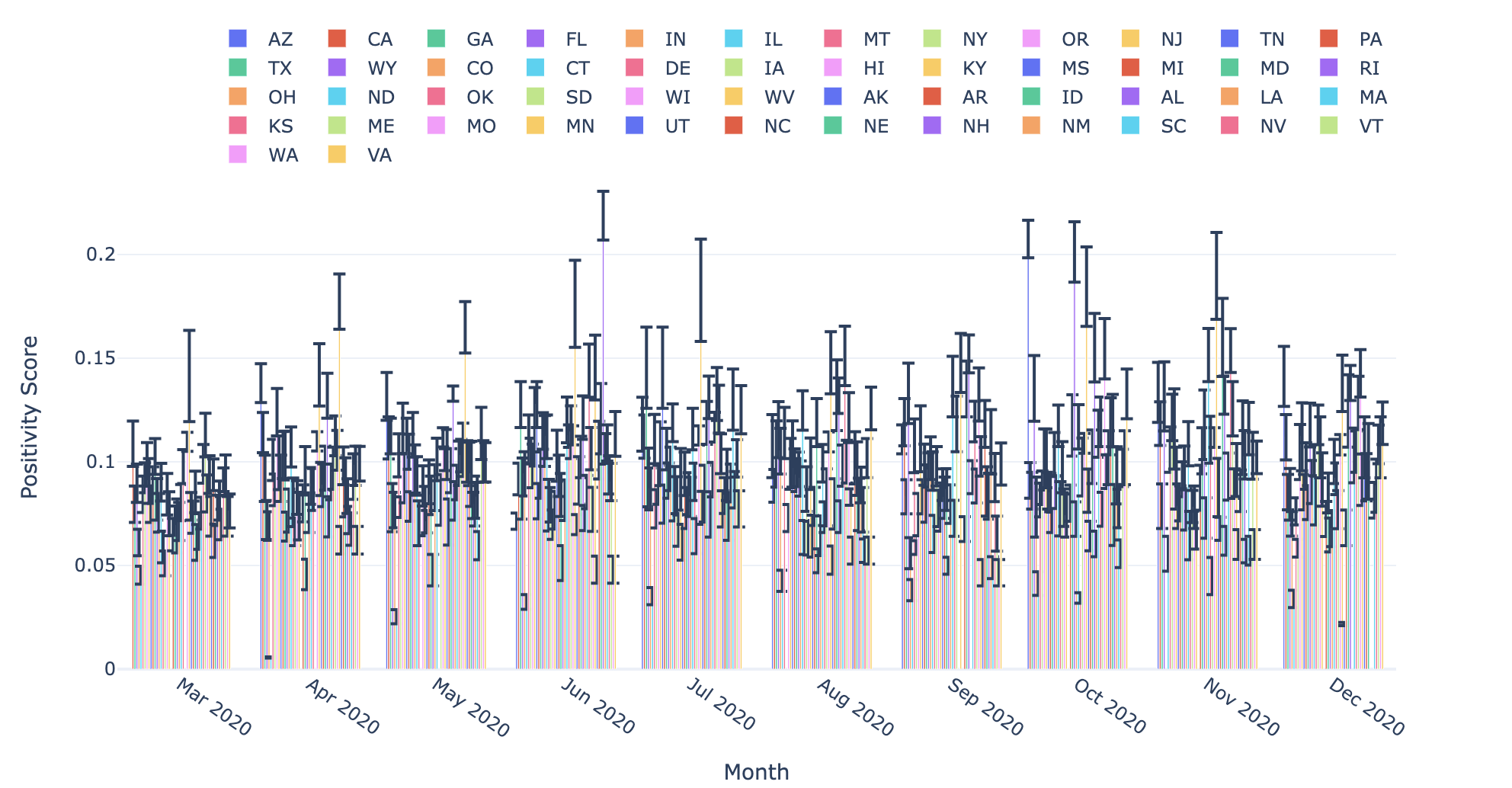


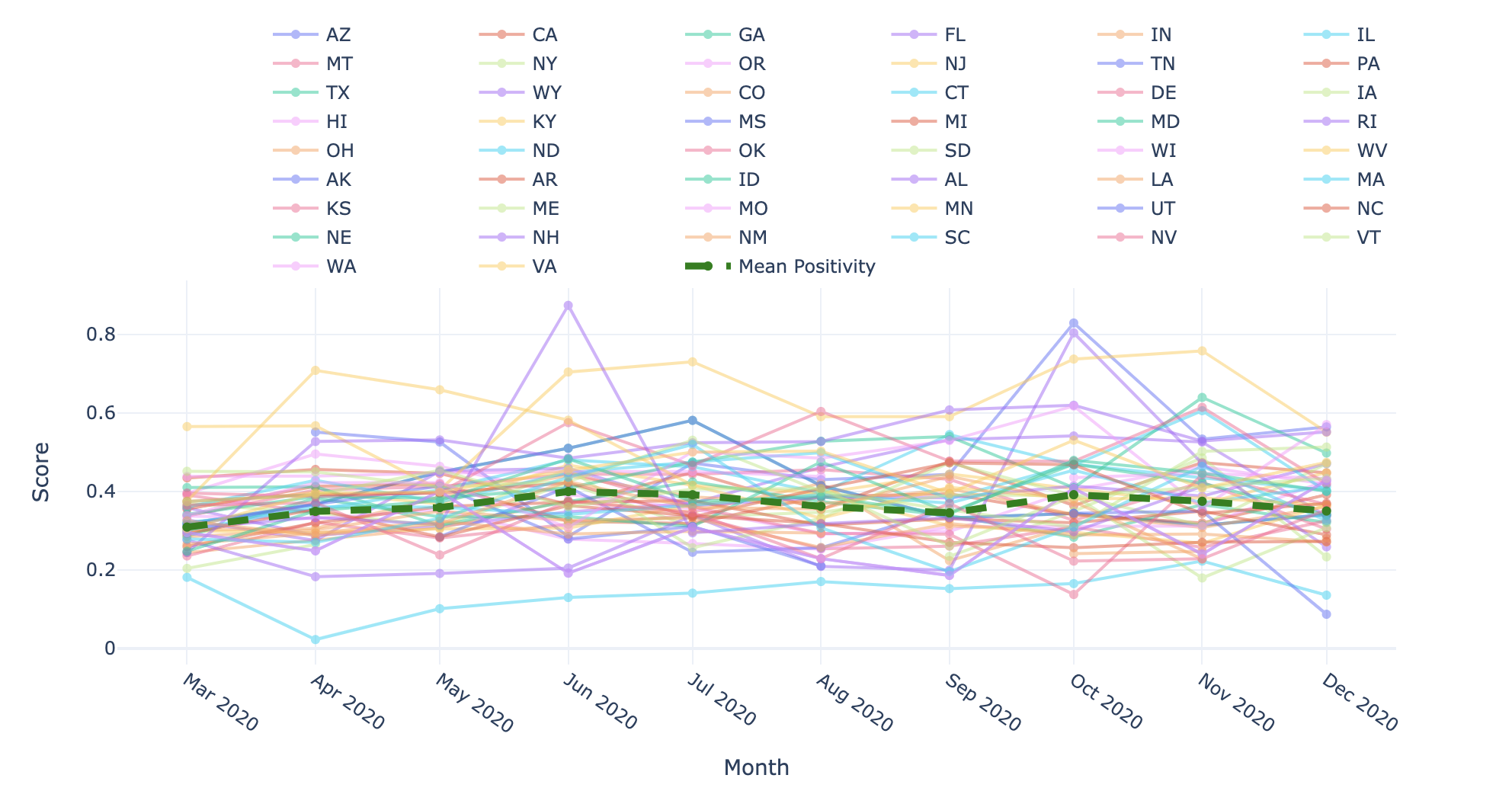

Supplement: S4 Appendix — (DOCX) [file pone.0272558.s004.docx]
